# Net ammonium and nitrate fluxes in wheat roots under different environmental conditions as assessed by scanning ion-selective electrode technique

**DOI:** 10.1038/srep07223

**Published:** 2014-11-27

**Authors:** Yangquanwei Zhong, Weiming Yan, Juan Chen, Zhouping Shangguan

**Affiliations:** 1 State Key Laboratory of Soil Erosion and Dryland Farming on the Loess Plateau, Northwest A&F University, Yangling, Shaanxi 712100, P.R. China

## Abstract

Wheat is one of the most important food crops in the world, its availability affects global food security. In this study, we investigated variations in NH_4_^+^ and NO_3_^-^ fluxes in the fine roots of wheat using a scanning ion-selective electrode technique in the presence of different nitrogen (N) forms, N concentrations, and pH levels as well as under water stress. Our results show that the fine roots of wheat demonstrated maximum NH_4_^+^ and NO_3_^−^ influxes at 20 mm and 25 mm from the root tip, respectively. The maximal net NH_4_^+^ and NO_3_^−^ influxes were observed at pH 6.2 in the presence of a 1/4 N solution. We observed N efflux in two different cultivars following the exposure of roots to a 10% PEG-6000 solution. Furthermore, the drought-tolerant cultivar generally performed better than the drought-intolerant cultivar. Net NH_4_^+^ and NO_3_^−^ fluxes may be determined by plant growth status, but environmental conditions can also affect the magnitude and direction of N flux. Interestingly, we found that NO_3_^−^ was more sensitive to environmental changes than NH_4_^+^. Our results may be used to guide future hydroponic experiments in wheat as well as to aid in the development of effective fertilisation protocols for this crop.

As an essential constituent of proteins, nucleic acids, chlorophylls and many secondary metabolites, nitrogen (N) is one of the major elements required for plant growth. Insufficient accumulation as well as the excess accumulation of N may compromise various plant functions. Ammonium (NH_4_^+^) and nitrate (NO_3_^−^) are two common forms of inorganic N that can serve as limiting factors for plant growth^1^,^2^.

To enable the performance of a variety of functions, the root system is composed of anatomically, morphologically and physiologically distinct root types that demonstrate a high degree of plasticity in terms of their responses to external signals and adaptation to heterogeneous nutrient supplies^3^,^4^. These anatomical and physiological complexities often determine the NH_4_^+^ and NO_3_^−^ absorption capacity of the root. NH_4_^+^ and NO_3_^−^ fluxes in roots have been investigated in many previous studies over the past few decades. Spatial and temporal variability in NH_4_^+^ and NO_3_^−^ uptake have been demonstrated along the lengths of roots in herbaceous and woody plants. The net flux of NO_3_^−^ appears to be low near the root apex and high in the basal regions of maize^5^ and barley roots^6^. However, in rice and carob seedlings, the opposite pattern has been reported^7^,^8^. A previous study of *Pinus pinaster* has shown that the highest NO_3_^−^ uptake rate occurs in an area 20–50 mm along the root axis from the root tip^9^. More recently, Luo, et al.^1^ have demonstrated marked spatial variability in NH_4_^+^ and NO_3_^−^ fluxes in the roots of the woody plant species *Populus popularis*.

NO_3_^−^ uptake is thought to be strongly regulated by a plant's demand for N^10^. The physiological mechanisms underlying the interactions between net NH_4_^+^ and NO_3_^−^ fluxes and the environment remain unclear. Hawkins, et al.^11^ have demonstrated that net NH_4_^+^ uptake is unaffected by the presence of NO_3_^−^ and vice versa in the roots of Douglas fir and lodgepole pine trees. However, the net uptake of NO_3_^−^ is markedly reduced in the presence of NH_4_^+^ in non-mycorrhizal roots of corn plants^12^ and *Pinus pinaster*^13^. NH_4_^+^ and NO_3_^−^ absorption share common pathways because both ions are actively absorbed by root cells at low external concentrations. Furthermore, NH_4_^+^ and NO_3_^−^ influx measurements have indicated the presence of two high-affinity transport systems (HATS) for NO_3_^−^ (one constitutive and the other inducible) and one HATS for NH_4_^+^^14^. However, the energetic and biochemical characteristics of NH_4_^+^ and NO_3_^−^ assimilation differ, resulting in differing net fluxes of these ions in roots as well as variable NH_4_^+^ or NO_3_^−^ preferences in some plants^15^. Many studies have shown that some species of boreal forest plants preferentially absorb NH_4_^+^ or amino acids over NO_3_^−^14,16,17, even when the concentration of NO_3_^−^ exceeds that of NH_4_^+^ by as much as 10-fold. In addition, the uptake of NH_4_^+^ has been shown to greatly exceed that of NO_3_^−^ in spruce tree roots but not in beech tree roots^18^. However, several plant species that have been supplied with moderate concentrations of NH_4_^+^ as the sole N source have shown reduced growth compared with their growth in the presence of similar amounts of NO_3_^−^19,20,21. This reduction in plant growth in the presence of NH_4_^+^ as the sole N source has been attributed to the combined effects of the acidification of the root zone^2^ and the toxic accumulation of free NH_4_^+^ or ammonia in plant tissues^22^,^23^. Rhizosphere pH affects the availability, uptake and assimilation of N ions by plants. Moreover, the temporal dynamics of net ion fluxes and the influences of other ions and environmental factors, such as pH, have been reported in the roots of maize, barley, rice, conifer and Eucalyptus species^5^,^7^,^24^,^25^,^26^,^27^. The temporal dynamics of net ion fluxes in roots in the presence of salinity stress have been widely studied, but few studies have examined these temporal dynamics under drought conditions^28^,^29^,^30^,^31^.

Wheat (*Triticum aestivum* L.) is one of the most important food crops in the world, and it plays an important role in global food security. Climate change and the use of urea can result in dry and acidified soil, which is detrimental to wheat crop yields. NH_4_^+^ and NO_3_^−^ are often used as wheat fertilisers to maximise crop yields. Therefore, information regarding the NH_4_^+^ and NO_3_^−^ fluxes in wheat roots exposed to various conditions (e.g., different forms of N (NH_4_^+^, NO_3_^−^ or both), varying pH levels and drought conditions) can be used to aid in the improvement of N fertiliser management practices in wheat farming.

Scanning ion-selective electrode technique (SIET) is an electrophysiological method that can non-invasively measure ion/molecule-specific activities^32^. To date, NH_4_^+^, NO_3_^−^, Ca_2_^+^, H^+^, Na^+^, K^+^, Cl^−^, Mg^2+^, Cd^2+^, Al^3+^ and O_2_ have been detected using SIET; however, its use for the examination of temporal and spatial patterns of net NH_4_^+^ and NO_3_^−^ fluxes in wheat roots exposed to different environmental conditions has not yet been reported.

In this study, we used SIET to investigate ion fluxes in wheat roots. Net NH_4_^+^ and NO_3_^−^ fluxes in fine roots of wheat that were exposed to different environmental conditions were measured non-invasively with SIET. The aims of this study were as follows: (1) to examine the spatial patterns of net NH_4_^+^ and NO_3_^−^ fluxes and to determine the locations relative to the root tips at which the maximal net uptake of these ions occurs in wheat; (2) to monitor alterations in net NH_4_^+^ or NO_3_^−^ fluxes in response to various environmental stimuli, including pH alterations, different N forms and N levels and drought stress; and (3) to assess the net NH_4_^+^ and NO_3_^−^ fluxes in the roots of two wheat cultivars and the differences in their responses to drought-like conditions. This study represents the first attempt to detect net NH_4_^+^ and NO_3_^−^ fluxes in wheat in the presence of various N forms, N concentrations and pH and under drought conditions using SIET. Our results may aid in the development of future hydroponic wheat experiments and effective fertilisation protocols for soil-grown wheat crops.

## Results

### Locations of maximal net NH_4_^+^ and NO_3_^−^ uptake

**Figure 1:**
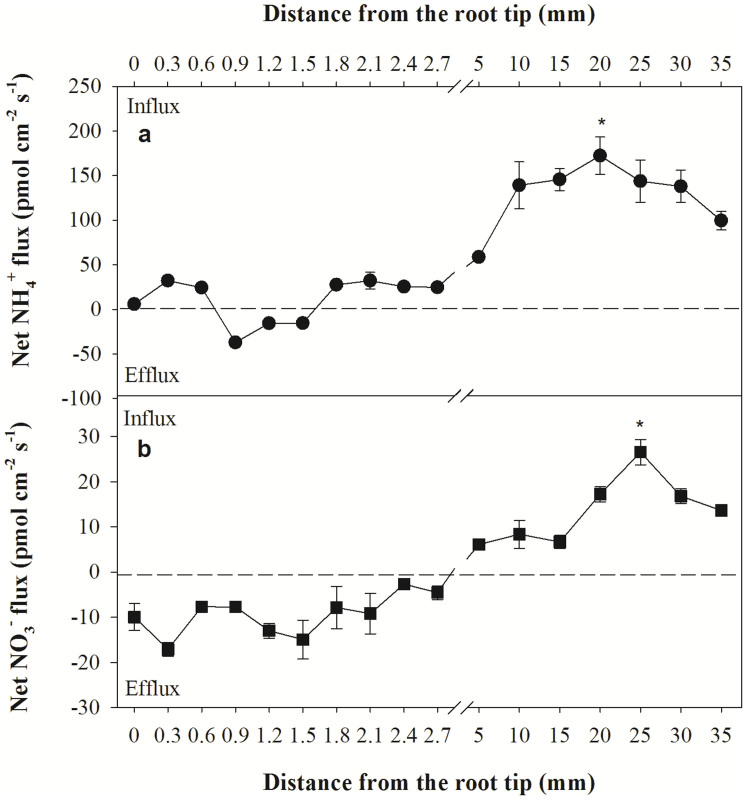
Net NH_4_^+^ (a) and NO_3_^−^ (b) fluxes along root tips of wheat. The data represent the mean ± SE (n = 6). Asterisks indicate significant differences between the measurements in question. Net influxes are suggested by positive values, whereas net effluxes are indicated by negative values.

To determine the areas along the root axis corresponding with maximal net NH_4_^+^ and NO_3_^−^ uptake, the net fluxes of these ions were measured along the root tips to an area located 35 mm from the apex (Fig. 1). These measurements widely varied at the different locations; for example, net NH_4_^+^ flux varied from −37.2 ± 2.6 (efflux) to 172.4 ± 21.0 (influx) pmol cm^−2^ s^−1^ along the root axis (Fig. 1a), whereas net NO_3_^−^ flux varied from −17.1 ± 1.5 (efflux) to 26.5 ± 2.7 (influx) pmol cm^−2^ s^−1^ (Fig. 1b). The maximum net NH_4_^+^ and NO_3_^−^ influxes occurred in an area between 20 mm and 25 mm from the root apex, respectively.

### Net NH_4_^+^ and NO_3_^−^ fluxes in the presence of different N forms

**Figure 2:**
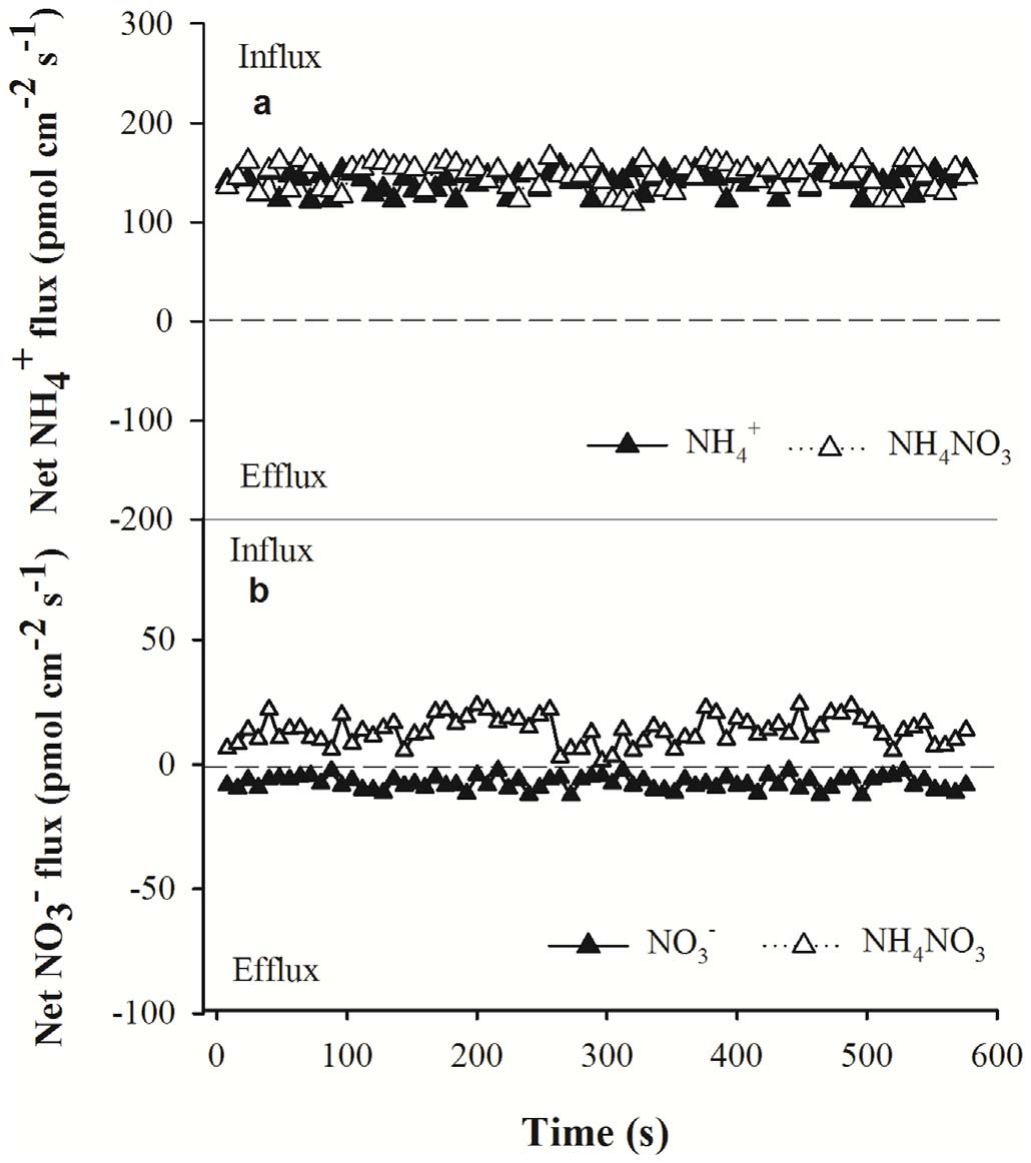
Net NH_4_^+^ (a) and NO_3_^−^ (b) fluxes over a period of 10 min in the fine roots of wheat incubated in NH_4_^+^, NO_3_^−^ and NH_4_NO_3_ solutions. The data represent the mean ± SE (n = 6). The mean fluxes of NH_4_^+^ and NO_3_^−^ during the measurement period are shown.

**Figure 3:**
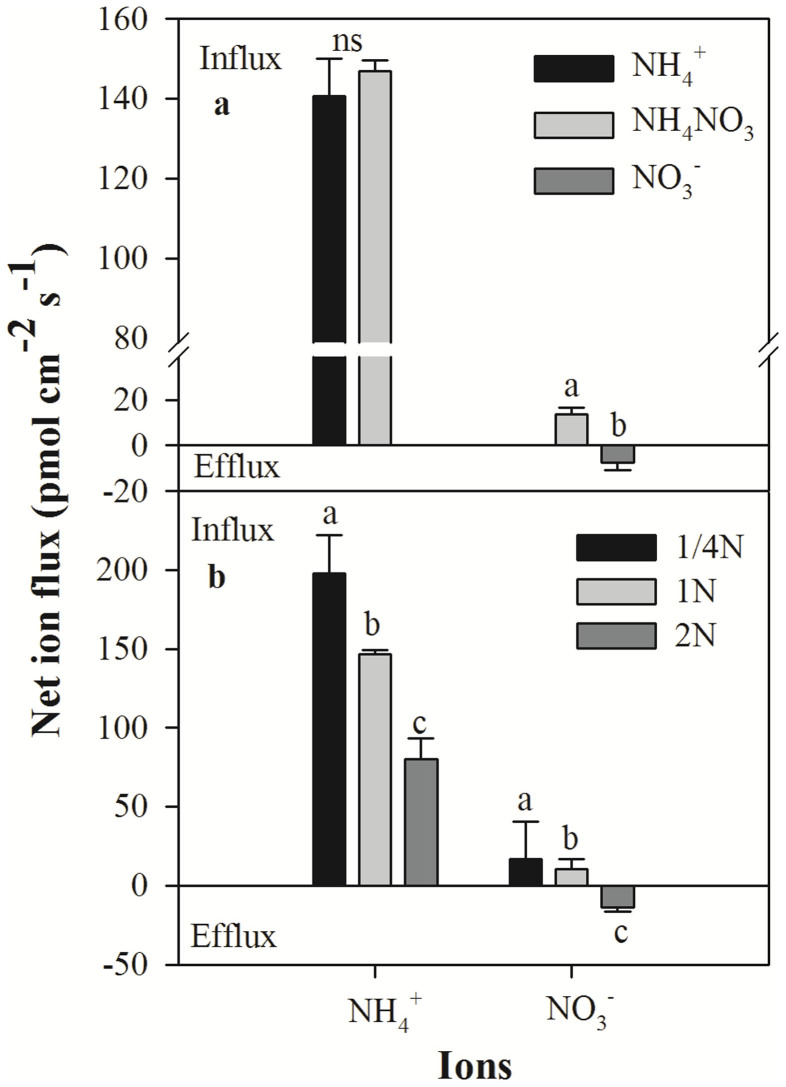
Net NH_4_^+^ and NO_3_^−^ fluxes under different environmental conditions. (a) NH_4_^+^ and NO_3_^−^ fluxes in the presence of different N sources; (b) NH_4_^+^ and NO_3_^−^ fluxes in the presence of different N concentrations. The data represent the mean ± SE (n = 6). The different letters indicate statistical significance at a p<0.05, and ns corresponds to a p>0.05.

At the locations corresponding with the highest net NH_4_^+^ and NO_3_^−^ influxes in the wheat roots, detailed measurements of the net fluxes of these ions were obtained (Fig. 2). Twenty millimetres from the root apex, slight fluctuations in net NH_4_^+^ were observed over a 10-min period (Fig. 2a). No significant differences were observed in net NH_4_^+^ fluxes in the roots exposed to NH_4_^+ ^and NH_4_NO_3_ solutions; the mean net NH_4_^+^ fluxes in the roots exposed to these solutions for 10 min were 140.6 ± 9.4 pmol cm^−2^ s^−1^ and 146.9 ± 2.7 pmol cm^−2^ s^−1^, respectively (Fig. 3a). However, 25 mm from the root apex, net NO_3_^−^ fluxes differed markedly in roots exposed to NO_3_^−^ and NH_4_NO_3_ solutions for 10 min (Fig. 2b). Following exposure to the NO_3_^−^ solution, the mean net NO_3_^−^ efflux was 7.5 ± 3.1 pmol cm^−2^ s^−1^, whereas following exposure to the NH_4_NO_3_ solution, the mean net influx of this ion was 13.8 ± 2.9 pmol cm^−2^ s^−1^ (Fig. 3a).

### Net NH_4_^+^ and NO_3_^−^ fluxes in response to different concentrations of NH_4_NO_3_ solution

The net NH_4_^+^ and NO_3_^−^ fluxes observed in wheat roots that were grown in solutions containing different levels of ammonium nitrate markedly differed (Fig. 3b). The maximum net NH_4_^+^ and NO_3_^−^ influxes in the presence of a 1/4 N solution were 198.0 ± 24.3 and 16.8 ± 23.1 pmol cm^−2^ s^−1^, respectively. The uptake rate of NH_4_^+^ by the roots was significantly higher than that of NO_3_^−^; however, this difference in uptake decreased as the concentration of the solution increased. The net NO_3_^−^ flux changes correlated with the net NH_4_^+^ flux changes; however, following treatment with a 2 N solution, NO_3_^−^ ions in the backfilling solution effluxed at a rate of 13.8 ± 2.3 pmol cm^−2^ s^−1^ (Fig. 3b).

### Net fluxes of NH_4_^+^, NO_3_^−^ and H^+^ at different pH levels

**Figure 4:**
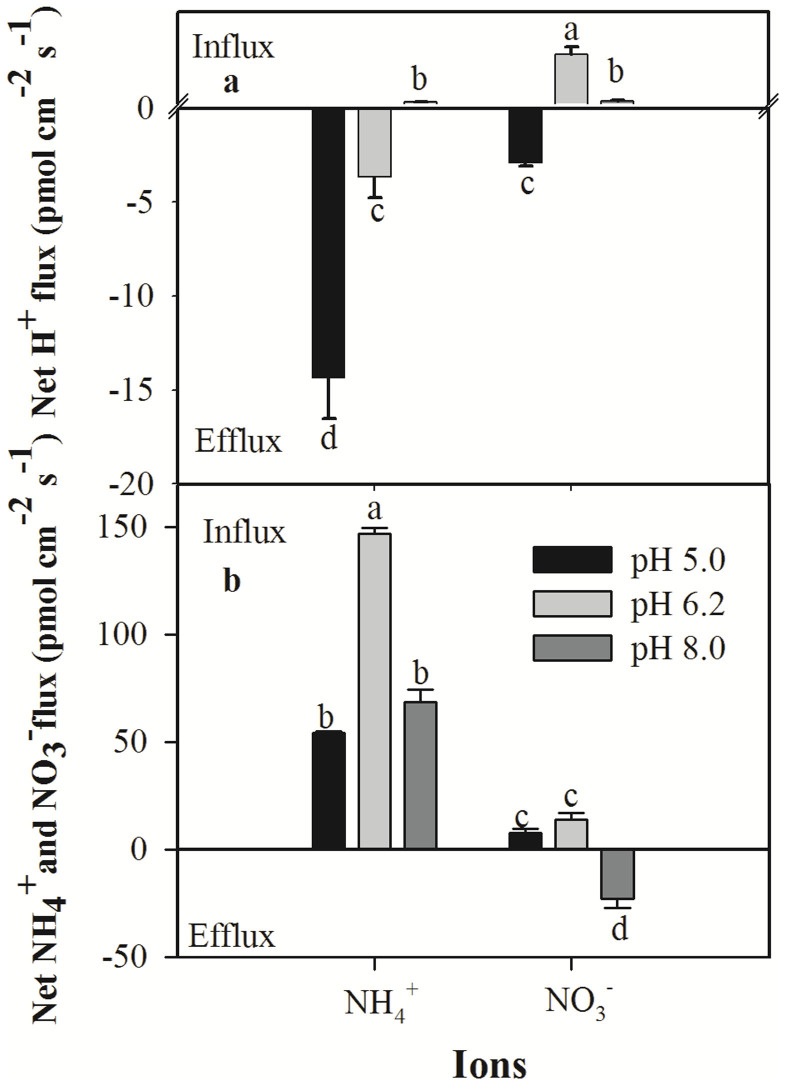
Net NH_4_^+^ and NO_3_^−^ fluxes at different pH levels and corresponding H^+^ fluxes at two sites along the root axis. The data represent the mean ± SE (n = 6). The different letters indicate statistical significance corresponding with a p<0.05.

Solution pH affects N and H^+^ uptake and assimilation by plants. In wheat roots, pH had a significant effect on net proton flux; net proton efflux was observed at pH 5.0, and net proton influx was observed at pH 8.0 (Fig. 4). The net efflux of H^+^ was the highest at pH 5.0, and the net flux of H^+^ at pH 8.0 was smaller than that observed at pH 6.2 (Fig. 4a). The net NH_4_^+^ and NO_3_^−^ fluxes in the wheat roots incubated at different pH levels also varied (Fig. 4b). The maximum net NH_4_^+^ and NO_3_^−^ influxes, which occurred at pH 6.2, were 146.9 ± 2.7 and 13.8 ± 2.2 pmol cm^−2^ s^−1^, respectively. The net influx of NH_4_^+^ did not differ at pH 5.0 and 8.0, and at all three pH levels, NH_4_^+^ exhibited a net influx. However, the roots displayed a net efflux of NO_3_^−^ at a rate of 23.1 ± 2.1 pmol cm^−2^ s^−1^ at pH 8.0, which was lower than the net influx of NH_4_^+^ (68.4 ± 2.9 pmol cm^−2^ s^−1^). The total influxes of N ions in the wheat roots were 61.7, 160.7 and 45.3 pmol cm^−2^ s^−1^ at pH levels of 5.0, 6.2 and 8.0, respectively (Fig. 4b).

### Net NH_4_^+^ and NO_3_^−^ fluxes under water stress

**Figure 5:**
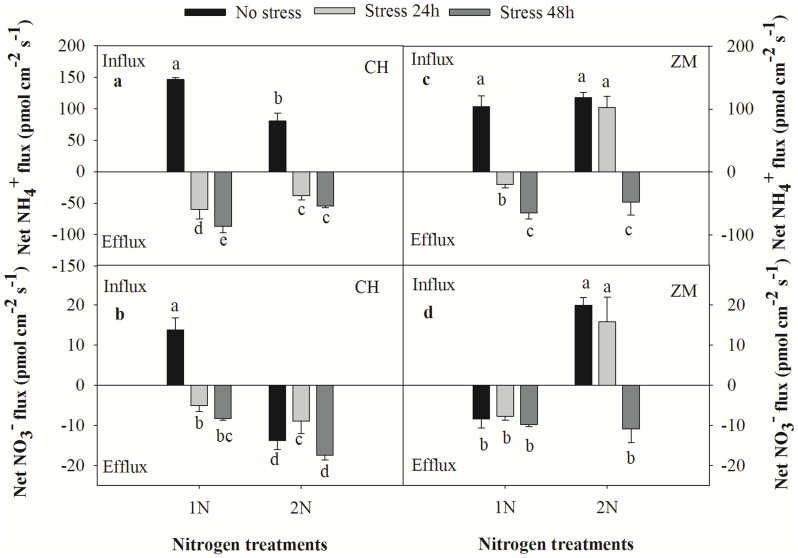
Net NH_4_^+^ and NO_3_^−^ fluxes under water stress in the two wheat cultivars in the presence of different N concentrations. PEG-6000 (10%, −0.32 MPa) was added to the nutrient solutions to simulate water stress. (a) and (b) correspond to the CH cultivar, whereas (c) and (d) correspond to the ZM cultivar. The data represent the mean ± SE (n = 6). The different letters indicate statistical significance corresponding to a p<0.05, and ns corresponds to a p>0.05.

Following exposure to water stress, the net flux of NH_4_^+^ in the wheat roots varied significantly between the two cultivars (Fig. 5). In the CH cultivar, NH_4_^+^ influx switched to efflux and the efflux rate increased in a time-dependent manner following exposure to water stress. However, we did not observe a statistically significant time-dependent difference in the efflux rate following 24 h versus 48 h of exposure to water stress. In the 2 N treatment group, the rate of net NH_4_^+^ flux was consistently lower compared with that of the 1 N treatment group. However, the net flux of NH_4_^+^ in the ZM cultivar exhibited some interesting differences. In the ZM and CH cultivar 1 N treatment groups, the net influx of NH_4_^+^ switched to efflux after 24 h of exposure to water stress. When the ZM cultivar was subjected to water stress in the presence of the 2 N solution, the switch to NH_4_^+^ and NO_3_^−^ efflux occurred after 48 h of stress exposure. The rate of net NH_4_^+^ efflux after 48 h of stress exposure in the presence of the 1 N solution was 87.0 ± 10.2 pmol cm^−2^ s^−1^ for the CH cultivar and 65.0 ± 9.6 pmol cm^−2^ s^−1^ for the ZM cultivar, whereas the net NH_4_^+^ efflux after 48 h of stress exposure in the presence of the 2 N solution was 54.2 ± 2.8 pmol cm^−2^ s^−1^ for CH and 47.6 ± 20.5 pmol cm^−2^ s^−1^ for ZM. Net NO_3_^−^ flux following exposure to the 1 N solution was similar to that of NH_4_^+^; NO_3_^−^ influx switched to efflux in the presence of water stress. The net NO_3_^−^ flux rates in the CH roots following treatment with the 1 N solution were 13.8 ± 2.9 (influx), −5.0 ± 1.4 (efflux) and −8.3 ± 0.4 (efflux) pmol cm^−2^ s^−1^. Net NO_3_^−^ flux in the presence of the 2 N solution was significantly different compared with that observed in the presence of the 1 N solution; the CH wheat roots that were unstressed, stressed for 24 h and stressed for 48 h exhibited NO_3_^−^ efflux rates of 13.8 ± 2.2, 9.0 ± 3.0 and 17.5 ± 1.1 pmol cm^−2^ s^−1^, respectively. NO_3_^−^ efflux in the ZM cultivar differed from that in the CH cultivar; the ZM cultivar exhibited efflux in the presence of the 1 N solution under no stress and after 24 h and 48 h of stress exposure. In the presence of the 2 N solution, NO_3_^−^ and NH_4_^+^ efflux occurred after 48 h of stress exposure.

## Discussion

### Variations in NH_4_^+^ and NO_3_^−^ fluxes along the root tip of wheat

Higher net NH_4_^+^ and NO_3_^−^ fluxes occurred in the white zone of wheat, which is located between 5 mm and 30 mm from the root tip. Previous studies have suggested that different zones of the root apical region exhibit distinct net fluxes of NH_4_^+^ and/or NO_3_^−^1,11,33,34. We observed that the spatial variability and net influxes of NH_4_^+^ and NO_3_^−^ were the highest at 20 and 25 mm from the root tips, respectively, in the fine roots of the wheat plants (Fig. 1).

Garnett, et al.^25^ have reported no consistent pattern of net NH_4_^+^ or NO_3_^−^ flux in an area located between 20 and 60 mm from the root tips of *E. nitens*; however, studies analysing several other plant species have shown variations in ion uptake rates along root axes. Seedlings of some woody plants show the highest net NH_4_^+^ and NO_3_^−^ uptake between 5 and 20 mm from root tips^1^,^11^. In 18–20-day-old rice plants, net NH_4_^+^ uptake declines in the more basal regions of the root, but maximal net NO_3_^−^ uptake occurs at 21 mm from the apex, declining thereafter^7^. Henriksen, et al.^5^ have reported that net NO_3_^−^ uptake increases with distance from the root tip up to 60 mm, whereas maximal net NH_4_^+^ uptake occurs in an area located between 10 and 20 mm from the root tip in 7-day-old barley. Different N ion uptake profiles may reflect differences in root anatomy and rates of root growth^35^, correlating with gene expression patterns and flux profiles along the lengths of young roots.

### NH_4_^+^ and NO_3_^−^ fluxes respond to environmental conditions

Pre-treatment may induce NO_3_^−^ and NH_4_^+^ transporter expression in roots of wheat seedlings as indicated by studies showing the substrate induction of root NO_3_^−^ and NH_4_^+^ transporters in many higher plants^36^,^37^. Our observations that the net influx of NH_4_^+^ was significantly higher than that of NO_3_^−^ in the roots incubated in the ammonium nitrate solution and that the maximal rate of N uptake occurred following concurrent exposure to NO_3_^−^ and NH_4_^+^ (Fig. 3) are consistent with previous studies of wheat^38^. Although the NH_4_^+^ concentration in the NH_4_^+^ solution was twice that in the ammonium nitrate solution, the net influx of this ion was not significantly different following the exposure of the roots to either solution, suggesting that the presence of NO_3_^−^ has a positive effect on net NH_4_^+^ uptake. These results are consistent with studies of wheat roots performed by Cramer and Lewis^39^. Interestingly, in the presence of NO_3_^−^ solution, the roots exhibited a net NO_3_^−^ efflux that was likely due to the dynamic balance of the influx and efflux of this ion at the root surface. We suspect that this net efflux in the presence of the NO_3_^−^ solution was largely determined by an increase in NO_3_^−^ efflux because high concentrations of this ion have been demonstrated to suppress its net influx and increase its efflux at the root surface^40^,^41^,^42^,^43^. In contrast, net NO_3_^−^ influx was observed in the roots incubated in the ammonium nitrate solution, suggesting that NH_4_^+^ did not interfere with NO_3_^−^ influx, whereas high concentrations of NO_3_^−^ appeared to inhibit the net uptake of this ion^40^. These results are in contrast with a previous study performed by MacKown, et al.^12^, in which NH_4_^+^ was shown to inhibit NO_3_^−^ uptake in corn.

**Figure 6:**
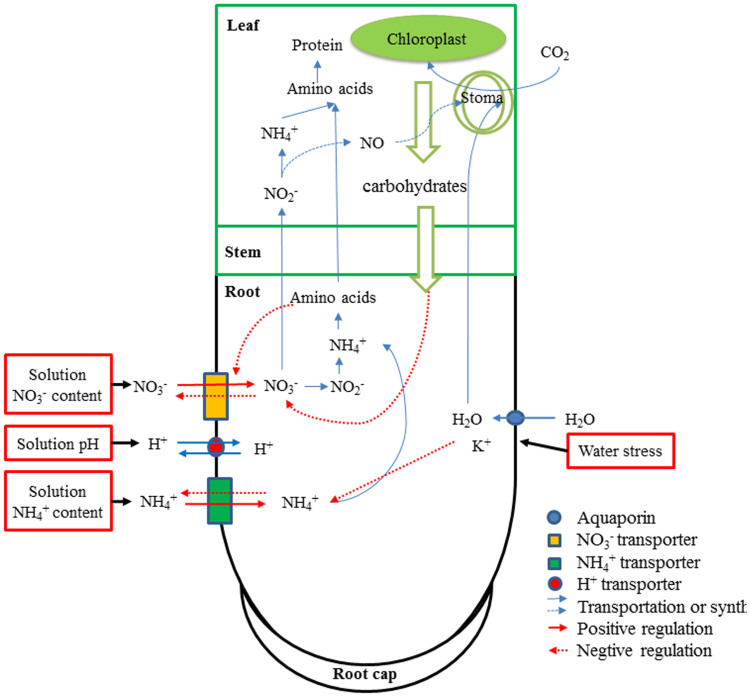
Proposed regulatory pathways for the efflux and influx of NH_4_^+^ and NO_3_^−^ in wheat. Maximum uptake of NH_4_^+^ occurs closer to the root tip than NO_3_^−^. NH_4_^+^ and NO_3_^−^ uptake are regulated by various environmental conditions and endogenous NH_4_^+^ and NO_3_^−^ concentrations. Furthermore, NH_4_^+^ and NO_3_^− ^uptake are driven by water flow due to transpiration^27^,^59^. pH value can affect N uptake based on the concentration of protons in the environment^27^. The NH_4_^+^ and NO_3_^−^ concentrations in a solution can also affect N flux. NO_3_^−^ can be converted to NH_4_^+^ by nitrate reductase (NR), after which NH_4_^+^ is converted to amino acids, whereas NH_4_^+^ taken up by plants can be converted directly to amino acids^59^. Excess amino acid accumulation negatively regulates NO_3_^−^ uptake, leading to the efflux of this ion, but NH_4_^+^ uptake is less affected by amino acid concentration. NO_3_^−^ is also transported to the leaf, and NO produced by NR can regulate stomatal aperture, leading to Pn and transpiration. Carbohydrates within roots that are produced by photosynthesis also help to regulate NO_3_^−^ influx^28^. When subjected to water stress, water loss and the regulation of K^+^ promote NH_4_^+^ and NO_3_^−^ efflux.

The highest rate of N uptake detected in the N-deprived plants was most likely due to the release of the roots from negative feedback, suggesting that the cytosolic concentrations of NH_4_^+^ and NO_3_^−^ were lower than the thresholds necessary for growth. The net rates of NH_4_^+^ and NO_3_^−^ uptake were the highest in the roots exposed to the 1/4 N solution followed by the 1 N solution and the 2 N solution. When NH_4_^+^ and NO_3_^−^ were supplied simultaneously, the roots exhibited a higher net influx or smaller net efflux of NH_4_^+^ compared with NO_3_^−^ (Fig. 1), but the magnitude of change differed according to the N concentration. The net NH_4_^+^ uptake was 12-fold greater than the net NO_3_^−^ uptake in the roots treated with the 1/4 N solution and was 14-fold greater in those treated with the 1 N solution. Similarly, net NH_4_^+^ uptake has been reported to be 2-fold greater than net NO_3_^−^ uptake at the maize root apex zone^7^ and 3-fold greater in rice roots^6^. Our data suggest that wheat roots exhibit a preference for NH_4_^+^ over NO_3_^−^, which may indicate that wheat seedlings require a greater uptake of NH_4_^+^ to meet the N demands necessary for rapid growth. There are several potential explanations for the observed preference for NH_4_^+^ influx compared with NO_3_^−^ influx. One reason may involve root morphology because different root tissues require different amounts of NH_4_^+^ and NO_3_^−^, and the meristem zone needs a higher concentration of NH_4_^+^ for protein synthesis^7^. In most species, NH_4_^+^ taken up by the roots is directly converted to amino acids within the roots, which cost less energy for both transport and assimilation (Fig. 6)^44^. Another reason that wheat roots prefer NH_4_^+^ to NO_3_^−^ is based on differences in the expression and activities of the transport systems for these ions in the different root zones. Net NH_4_^+^ and NO_3_^−^ uptake can be mediated by high-affinity transporters and by various low-affinity transporters. Furthermore, the uptake of these ions can be reversed by their efflux systems^45^, and several high-affinity NH_4_^+^ and NO_3_^−^ transporters have been cloned^46^,^47^. Britto et al. and Class et al.^14^,^48^, reproted that when high-affinity NH_4_^+^ fluxes are effectively regulated, transport via the low-affinity system is poorly regulated, this may resulting in considerable futile cycling of NH_4_^+^ across the plasma membrane as well as toxic effects of excessive NH_4_^+^ accumulation. In our study, NO_3_^−^ are more variable in different enviroment conditions. This may be explained by that NO_3_^−^ is able to function both as an osmoticum and as a mobile ion as Salsac, et al.^49^ reported. In all, the changes for NH_4_^+^ and NO_3_^−^ in different solutions may be explained by these ion characteristics and regulation mechanisms in wheat.

The net NH_4_^+^ and NO_3_^−^ influxes appeared to be the highest at pH 6.2, which would presumably result in the fastest growth of the wheat. Exposure to low and high pH levels resulted in relatively lower net NH_4_^+^ and NO_3_^−^ uptake in the wheat roots (Fig. 4). The differences in NH_4_^+^ and NO_3_^−^ uptake in response to pH may be related to the ability of wheat roots to maintain proton efflux (Fig. 6), as indicated by previous studies suggesting that H^+^ may be co-transported along with cations, such as NH_4_^+^^50^, and anions, such as NO_3_^−^1,27. Roots that absorb N in the form of NO_3_^−^ tend to exhibit a decrease in proton efflux, resulting in an increase in pH within the rhizosphere, whereas roots that absorb NH_4_^+^ tend to show an increase in proton efflux, which leads to a lower pH in the rhizosphere^51^,^52^. Due to the importance of protons in the regulation of N uptake and assimilation, the differences in proton flux in the presence of various pH levels that were observed in this study are intriguing. Previous studies have shown that plants grown at a low pH show an increase in H^+^-ATPase protein activity and maintain a high rate of proton efflux as a means to acclimate to acidic environments^27^,^53^,^54^. Changes in H^+^ concentration due to pH treatment could have affected H^+^-ATPase activity, resulting in significant changes in H^+^ flux from the root cells, indirectly affecting N flux. The low rate of NO_3_^−^ influx at pH 5.0 could also have been due to negative effects of the high chloride ion concentration on NO_3_^− ^transporters because these two anions have been shown to compete for the same transporter^55^. The influence of pH on N ion uptake is complex; thus, we are not surprised that results vary among studies investigating this phenomenon^27^,^56^.

We observed a net influx of NO_3_^−^ in the roots in the presence of the 1 N solution, which changed to efflux in the presence of the 2 N solution in the drought-tolerant CH cultivar. These findings were completely opposite of those observed in the water-sensitive ZM cultivar. However, we detected a net influx of NH_4_^+^ in the roots of both cultivars. The net NH_4_^+^ uptake in the presence of the 1 N solution was higher than that in the presence of the 2 N solution for the CH cultivar. Moreover, no differences in net NH_4_^+^ uptake were observed in the roots of the ZM cultivar exposed to non-stress conditions, which may have been due to differences in genotypes (Fig. 5). However, following exposure to 10% PEG, we observed N efflux after additional treatments with the 1 N and 2 N solutions for 24 h and 48 h. When CH was exposed to the 2 N solution and water stress for 24 h, NH_4_^+^ efflux was observed. When this cultivar was treated with the 1 N solution, NH_4_^+^ efflux was higher than that observed following treatment with the 2 N solution, and these results were the opposite of those obtained with the CH cultivar in terms of NO_3_^−^ flux. In the ZM cultivar, no differences in N efflux were observed after 48 h of water stress in the presence of either solution (Fig. 5). Plant growth responds to drought stress with rapid, osmotic changes that parallel those that occur following salinity stress^31^. Drought stress leads to water loss or a reduction in water absorption by roots. This can cause disturbances in the mineral nutrient balances of plants and can also lead to ion deficiencies or other nutrient imbalances due to the competition of nutrients for various cations and anions^29^. The influence of drought stress on N ion uptake is very complex. Our study is the first to examine net N flux using SIET, and our results suggest that N efflux represents a drought stress response involving nutrient efflux aimed at decreasing the plant growth rate. In addition, the net efflux of NO_3_^−^ and NH_4_^+^ may also be influenced by the influx or efflux of other ions, such as K^+^ and Ca^2+^, which play important roles in drought and salt stress (Fig. 6)^29^,^30^,^31^. The net N uptake in the 2 N solution was lower than that of the 1 N solution, suggesting that extra nutrition may alleviate the detrimental effects of drought. These results are consistent with studies showing that increasing the supply of nutrients to plant growth media maintained under drought-like conditions can alleviate the adverse effects of drought on plant growth^29^. The response of the CH cultivar to the drought-like conditions was more rapid than that of ZM, revealing that the rapid efflux of N was able to slow the growth rate and prevent additional drought-induced damage from occurring. We suspect that this ability of CH permits it to perform better than ZM under similar drought conditions as indicated by our previous study^57^.

Overall, the simultaneous uptake and assimilation of NO_3_^−^ and NH_4_^+^ in the wheat roots was influenced by the endogenous N concentration and exogenous supply of substrates (Fig. 6)^2^,^14^,^36^,^58^. The net N flux represents a balance of influx and efflux that is influenced by many factors, including soluble carbohydrates in the root, which can supply energy for NO_3_^−^ uptake by respiration^28^. Other factors that influence N flux include transporters that regulate N uptake^59^, the expression of high-affinity N transport systems^14^, the H^+^ concentration in the growth medium^27^, water flux^59^ and the fluxes of other ions^29^,^30^,^31^. Net NH_4_^+^ and NO_3_^−^ fluxes respond to environmental conditions differently according to plant growth status. To date, many studies of inorganic N uptake at the physiological and molecular levels have focused on the regulation of root plasma membrane transporters. Future physiological and molecular studies will be required to fully elucidate the mechanisms of N uptake that occur in plants.

## Conclusions

The elucidation of the mechanisms associated with N transport by evaluating net N flux is challenging. Net N flux is based on the sum of N influx and efflux, and it is influenced by the rates of assimilation and compartmentalisation^27^. Our results indicated that at the four-leaf stage, the maximum influxes of NH_4_^+^ and NO_3_^−^ occurred in an area between 20 mm and 25 mm from the root apex, respectively, in the fine roots of wheat. Interestingly, we found that NO_3_^−^ flux was more sensitive to environmental changes than that of NH_4_^+^. Furthermore, the wheat grown under optimal conditions absorbed more overall N, but this absorption was influenced by the form and concentration of N, the pH and the presence of water stress. Because the SIET method was used to measure the net fluxes of NH_4_^+^, NO_3_^−^ and H^+^ and not their individual rate of influx or efflux in the roots, further research is necessary to understand the biological implications of stoichiometric proportions of net NH_4_^+^, NO_3_^−^ and H^+^ fluxes in relation to environmental conditions. These results may aid in the elucidation of mechanisms associated with N uptake by roots and provide additional information with regard to the spatial and temporal patterns of net N uptake in wheat. Our findings may also be used to guide future hydroponic experiments with wheat and to develop effective fertilisation protocols for field-grown wheat.

## Methods

### Plant materials and treatments

Wheat (*Triticum aestivum* cv. Changhan No. 58 and Zhengmai No. 9023) seeds were obtained from Northwest A&F University (Yangling, Shaanxi, China), disinfected with 20% (w/v) sodium hypochlorite for 30 min to prevent fungal infection, rinsed with distilled water and placed on wet filter paper at 25°C for approximately 24 h in the dark. The cultivar Zhengmai No. 9023 (ZM) was water-sensitive and drought-intolerant, whereas the cultivar Changhan No. 58 (CH) was drought-tolerant and therefore suitable for drought-prone environments. The thousand-kernel weights of ZM and CH were 43.58 and 43.61 g, respectively. After the seeds sprouted, they were germinated in large petri dishes lined with moistened filter paper in an illuminated incubator at 25°C under a 12 h-12 h light-dark cycle. On the 7th day of germination, which is when the wheat plants had grown to the one-leaf stage, the seedlings were hydroponically cultured in 1/2 modified Hoagland nutrient solution in a closed-climate chamber (AGC-D001P, Qiushi Corp., China) under an 11 h dark period (18°C, RH 50%) and 13 h light period (25°C, RH 50%, 300 μmol photons m^−2^ s^−1^ from 6:30 a.m. to 7:30 p.m.). Nine wheat plants were cultivated in a 15 × 10 × 8 cm container filled with 1 L of nutrient solution that was aerated with an aquarium diffuser.

After two days of growth in 1/2 Hoagland nutrient solution, the nutrient solution was replaced with a treatment solution. Single-factor controlled experiments were designed to test the effects of pH (5.0, 6.2 and 8.0), N source (NH_4_^+^ and NO_3_^−^) and N concentration (1/4 N, 1 N, 2 N) on the CH cultivar. The ZM cultivar was grown only in the 1 N and 2 N solutions to determine the effects of water stress. Each treatment was repeated in three independent trials, and each trial included 9 wheat plants. The 1 N concentration of Hoagland nutrient solution consisted of 7.5 mM NH_4_NO_3_, 1 mM KH_2_PO_3_, 5 mM KCl, 5 mM CaCl_2_ and 2 mM MgSO_4 _for the CK cultivar; 7.5 mM (NH_4_)_2_SO_4_ was used in place of NH_4_NO_3_ for the NH_4_^+^ treatment condition. Furthermore, 5 mM Ca(NO_3_)_2_ and 5 mM KNO_3_ were used in place of NH_4_NO_3_, KCl and CaCl_2_ for the NO_3_^−^ treatment condition. For the 1/4 N and 2 N Hoagland solutions, NH_4_NO_3_ concentrations of 1.875 mM and 15 mM were used, respectively. The pH of the nutrient solution was verified using a pH meter. The nutrient solution was refreshed each day to prevent dilution. The wheat plants had grown to the four-leaf stage at 10 days after the initiation of the treatment, at which point the ion concentrations were measured. PEG-6000 (10% solution, -0.32 MPa) was added to the 1 N and 2 N Hoagland solutions of the CH and ZM cultivars, after which the plants were grown for an additional 24 h or 48 h.

### Measurement of ion flux at the root surface

To monitor the net fluxes of NH_4_^+^, NO_3_^−^ and H^+^ in wheat roots in response to pH alterations, white fine roots of wheat were selected and excised from the root system of each plant in each treatment group. The excised roots were immersed in a measuring solution (A: 0.1 mM KNO_3_, 0.1 mM KCl, 0.1 mM CaCl_2_ and 0.3 mM MES, pH 6.2; B: NH_4_^+^: 0.1 mM NH_4_Cl, 0.1 mM KCl, 0.1 mM CaCl_2_ and 0.3 mM MES, pH 6.2; C: NH_4_NO_3_: 0.1 mM NH_4_NO_3_, 0.1 mM KCl, 0.1 mM CaCl_2_ and 0.3 mM MES at pH 5.0, pH 6.2 or pH 8.0). MES refers to 2-(N-morpholino) ethanesulfonic acid hydrate buffer. Six of the most similar roots (two plants from each trial) from the NH_4_^+^ treatment group and from the NO_3_^−^ treatment group were used for ion flux analyses. Net ion flux was measured using the SIET technique (BIO-003A system; Younger USA Science and Technology Corp.; Applicable Electronics Inc.; Science Wares Inc., Falmouth, MA, USA), which was conducted on-site at Xuyue Science and Technology Co., Ltd. (Beijing, China). The SIET system and its application in ion flux detection have been described previously in detail^32^,^33^,^60^. Briefly, ion-selective microelectrodes designed with 2–4-μm apertures were manufactured and silanised (for the NH_4_^+^ electrode, 100 mM NH_4_Cl was used as a backfilling solution, followed by an NH_4_^+^-selective liquid ion exchange cocktail (#09879, Sigma); for the NO_3_^−^ electrode, 10 mM KNO_3_ was used as the backfilling solution, followed by an NO_3_^−^-selective liquid ion exchange cocktail (#72549, Sigma); for the H^+^ electrode, 15 mM NaCl and 40 mM KH_2_PO_4 _were used as the backfilling solutions, followed by an H^+^-selective liquid ion exchange cocktail (#95293, Sigma)). Prior to performing the flux measurements, the microelectrodes were calibrated (for the NH_4_^+^ measurements, 0.05, 0.5 and 0.1 mM NH_4_Cl in addition to other compounds were used for calibration; for the NO_3_^−^ measurements, 0.05, 0.5 and 0.1 mM KNO_3_ in addition to other compounds were used for calibration; for the H^+^ measurements, pH 5.0, 6.2, and 8.0 solutions in addition to other compounds were used for calibration). The calibration curves are shown in Supplemental Figure S1, and only electrodes with Nernstian slopes of higher than 55 mV per ten-fold concentration difference were used.

To determine the areas along the root where the maximal ion influxes of NH_4_^+^ and NO_3_^−^ occurred, a preliminary experiment was conducted, in which an initial measurement was performed at the root tip, followed by additional measurements in either 300-μm (between 0 and 2,700 μm from the root tip) or 5-mm (between 5 ± 1 and 35 ± 1 mm from the root tip) increments (Fig. 1). When maximal ion influxes were achieved, the fluxes of NH_4_^+^ and NO_3_^− ^were measured for each treatment. H^+^ concentration was measured in a similar area as the NH_4_^+^ and NO_3_^−^ concentrations to evaluate the pH treatments. Ion gradients (NH_4_^+^, NO_3_^−^ and H^+^) close to (approximately 5 μm above) the root surface were measured by moving the ion-selective microelectrode between two positions (separated by a distance of 30 μm) in a direction perpendicular to the root axis. The recording rate of ion flux was one reading per 6 s. Ion flux was recorded at each measurement point for 10 min. The amplifier curves generated by the measurements and representative images of real-time flux are shown in Supplemental Figures S2 and S3. Acquisition of root images was performed using Mageflux software (version 1.0) in association with the SIET system.

### Data processing and statistical analysis

Net ion flux data were calculated and exported using Mageflux software (version 1.0) in association with the SIET system^32^. For analyses of maximal net NH_4_^+^ and NO_3_^−^ fluxes, the net fluxes of these ions were measured within 10 min of each treatment, and the values were averaged. All statistical analyses were performed using SPSS software version 17.0 (SPSS Inc., Chicago, IL, USA). One-way ANOVA was performed to determine the significance of the differences observed. Significant differences were evaluated at a 95% confidence level. When significance was observed at p<0.05, a least significant difference (LSD) post hoc test was performed for multiple comparisons.

## Author Contributions

Y.Z., W.Y., J.C. and Z.S. conceived and designed the experiments. Y.Z. and W.Y. performed the experiments. Y.Z. and W.Y. analysed the data. Y.Z., W.Y., J.C. and Z.S. wrote the paper. All authors read and approved the final manuscript.

## Supplementary Material

Supplementary Information Supplementary Material
