# Supplementary material for: Net ammonium and nitrate fluxes in wheat roots under different environmental conditions as assessed by scanning ion-selective electrode technique
Source: Sci Rep. 2014 Nov 27;4:7223. doi: 10.1038/srep07223 (PMC4245524; doi:10.1038/srep07223)
Supplement: Supplementary Information — Supplementary Material [file srep07223-s1.doc]

**Title page**

**Title:** Net ammonium and nitrate fluxes in wheat roots under different environmental conditions as assessed by scanning ion-selective electrode technique

**Running title:** Net fluxes of ammonium and nitrate in wheat roots

**Authors’ names:** Yangquanwei Zhong^#^, Weiming Yan^#^, Juan Chen, Zhouping Shangguan*

^#^These authors contributed equally to this work and should be considered as co-first authors.

**Authors’ institution:** State Key Laboratory of Soil Erosion and Dryland Farming on the Loess Plateau, Northwest A&F University, Yangling, Shaanxi 712100, P.R. China

***Corresponding author:** Prof. Zhouping Shangguan

**Address:** Xinong Rd. 26, Institute of Soil and Water Conservation, Yangling, Shaanxi, 712100, P.R. China

Phone: ++86-29-87019107

Fax: ++86-29-87012210

E-mail: [shangguan@ms.iswc.ac.cn](mailto:shangguan@ms.iswc.ac.cn)

**Supplementary Material**

**Supplemental Figure S1 Calibration curves of NH_4_^+^, NO_3_^-^  and H^+^ microelectrodes.** Data represent the mean ± SE (n=3). The slopes represent Nernstian slopes, only electrodes with Nernstian slopes higher than 55 mV per ten-fold concentration difference were used.

**Supplemental Figure S2 Amplifier curves for the measurement of net NH_4_^+^ or NO_3_^-^ fluxes.** (a) NH_4_^+^ amplifier curves in NH_4_^+^ solution; (b) NH_4_^+^ amplifier curves in NH_4_NO_3_ solution; (c) NO_3_^-^ amplifier curves in NO_3_^-^ solution; (d) NO_3_^-^ amplifier curves in NH_4_NO_3_ solution; Data represent the mean(n=6).

**Supplemental Figure S3** **Representative images showing real time flux measurements of net NH_4_^+^ or NO_3_^-^  fluxes.** (a) NH_4_^+^ influx in NH_4_^+^ solution; (b) NH_4_^+^ influx in NH_4_NO_3_ solution; (c) NO_3_^-^ efflux in NO_3_^-^ solution; (d) NO_3_^-^ influx in NH_4_NO_3_ solution.


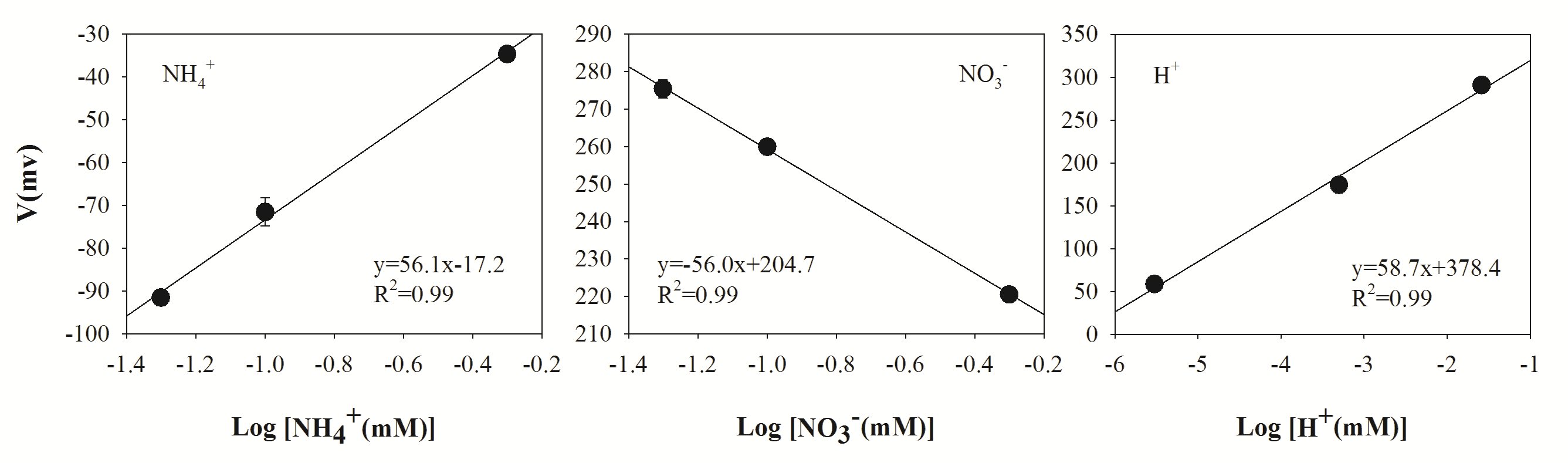


**Figure S1**


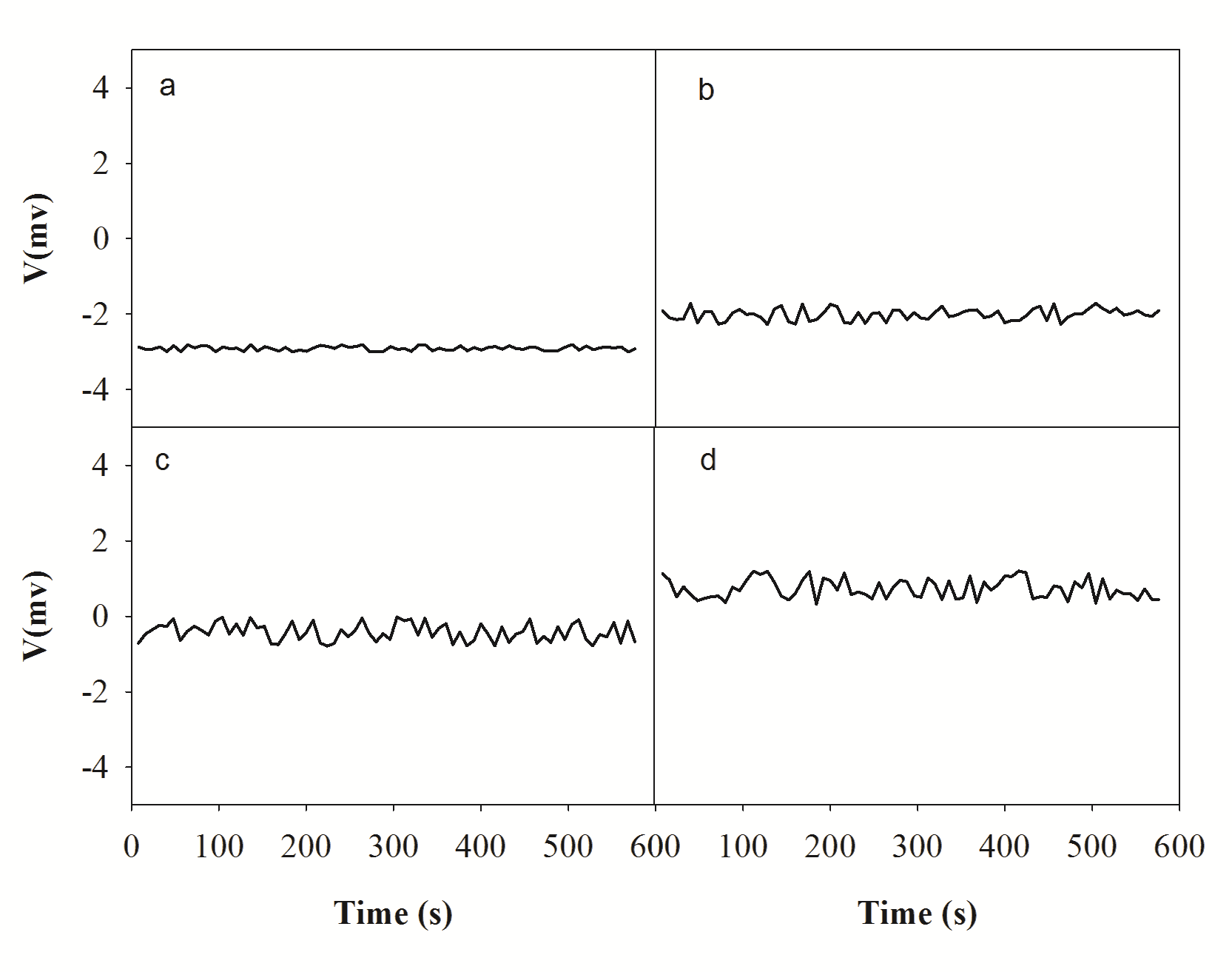


**Figure S2**


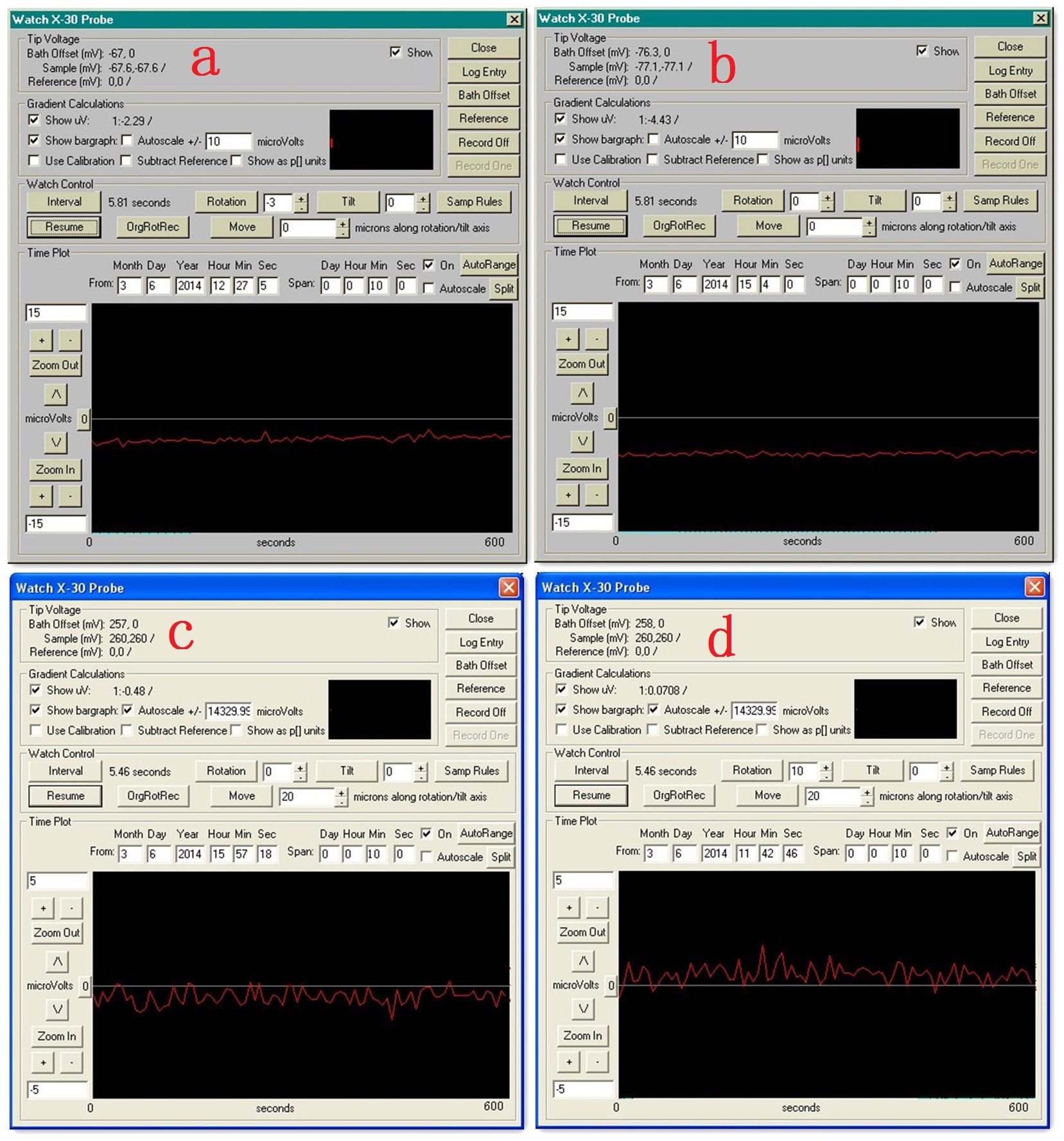


**Figure S3**
